# Comparison of Neural Network and Logistic Regression Analysis to Predict the Probability of Urinary Tract Infection Caused by Cystoscopy

**DOI:** 10.1155/2022/5775447

**Published:** 2022-03-21

**Authors:** Tsai-Jung Chen, Yu-Huang Hsu, Chieh-Hsiao Chen

**Affiliations:** ^1^Institute of Biochemical Technology National Chiayi University, Taiwan; ^2^Department of Urology, China Medical University and Beigang Hospital, Taiwan

## Abstract

**Purpose:**

Urinary tract infections (UTIs) are the most common infections among hospitalized patients. Cystoscopy is a minimally invasive procedure to check bladder disease, among the patients receiving procedure, approximately 10% of patients may experience UTI. In this study, a neural network model with high accuracy, sensitivity, and specificity was developed to predict the probability of UTIs caused by cystoscopic procedures. To reduce antibiotic overuse during cystoscopic procedures, the model can provide clinicians with a rapid assessment of whether patients require prophylactic antibiotics.

**Materials and Methods:**

Patients who underwent cystoscopic procedures at China Medical University Beigang Hospital from 2016 to 2019 were retrospectively reviewed. A total of 1647 patients were enrolled, and 147 cases of urinary tract infection occurred. An artificial neural network (ANN) and logistic regression analysis were used to develop the prediction models, and the two models were compared.

**Results:**

The logistic regression analysis model had an accuracy of 91%, sensitivity of 2%, and specificity of 99%, indicating that the logistic regression model predicted that most patients had a low risk of infection. The neural network model had a high accuracy of 85%, sensitivity of 80%, and specificity of 88%.

**Conclusions:**

Because the logistic regression model had low sensitivity and missed most cases of UTI, the logistic regression model is inappropriate for clinical application. The neural network model has superior predictive ability and can be considered a tool in clinical practice.

## 1. Introduction

Urinary tract infections (UTIs) are prevalent globally. Approximately 150 million people worldwide experience UTIs every year, causing a heavy burden on the economy and public health and considerably affecting patient quality of life [1, 2].

UTIs are a common complication of cystoscopy. Cystoscopy is an internal visual examination of the bladder and urethra [3]. Existing acute inflammation in the urinary system is likely to cause the spread of inflammation after a cystoscopic examination. Cystoscopy is mainly employed to examine the lower urinary tract, whereas physicians use endoscopy to identify the cause of UTI, SUI, or tumor. Cystoscopy is generally considered a safe and well-tolerated routine examination. Bacteria typically collect at the urethral meatus and anterior urethra. During cystoscopy, these bacteria may stick to the transurethral device. UTIs that occur after cystoscopy must be treated seriously. Although cystoscopy is a minimally invasive examination, it can lead to UTI in approximately 10% of patients [4, 5].

According to the Taiwanese guidelines for the use of prophylactic antibiotics in surgical operations, proper use of prophylactic antibiotics can reduce various postoperative infections. A lack of consensus on the use of prophylactic antibiotics in urinary tract examination and the increase in the number of drug-resistant bacterial strains have caused the misuse of antibiotics. Globally, more than 100,000 people die annually from drug-resistant infections [6]. Drug-resistant UTIs not only threaten the health and lives of patients but also increase burdens on family and social costs. Therefore, more attention to this problem is warranted.

Data mining has become a popular research field. The goal of data mining is to extract information from a database and transform it into an understandable structure for further use. In addition to the initial analysis steps, data mining involves database and data management; data preprocessing, modeling, and inference; and complexity considerations. Its purpose is to identify hidden and potentially useful information in unorganized big data.

This study adopted machine learning for data exploration. In the past, medical disease judgments were mostly based on physician experience. However, current disease judgment indicators are diverse. Diagnostic accuracy can be improved if these disease judgment indicators can be integrated with data mining. An accurate predictive system in clinical practice can provide urologists with highly precise assessments of whether patients undergoing cystoscopy require prophylactic antibiotics to reduce secondary infections and antibiotic abuse. An artificial neural network (ANN) and logistic regression analysis were the selected predictive tools in the present study. Both methods were used to predict the probability of UTI caused by cystoscopy, and the two methods were compared in terms of their predictive power.

## 2. Materials and Methods

Clinical data were collected on patients who underwent cystoscopy at China Medical University Beigang Hospital during 2016–2019. Among the medical records of 1647 patients, 147 cases of UTI within 1 month after undergoing cystoscopy were recorded. Only patients who underwent cystoscopy because of the presented symptoms were included; patients who underwent cystoscopy to remove a double-J stent were excluded. Variables in the collected data are presented in Table 1, and the standardized codes for the selected variables are listed in Table 2.

Microsoft Azure Machine Learning Studio (Microsoft, US) was used to establish the ANN prediction model. Microsoft Azure Machine Learning Studio is an artificial intelligence application in which drag-and-drop tools can be used to build, test, and predict analytical solutions to data. For logistic regression analysis, IBM SPSS Modeler (IBM, US) was used for advanced statistical analysis. These two programs were used to aggregate and analyze relevant data from a large number of data to develop the most suitable predictive models for assisting clinicians in predicting the probability of UTI caused by cystoscopy.

**Figure 1:**
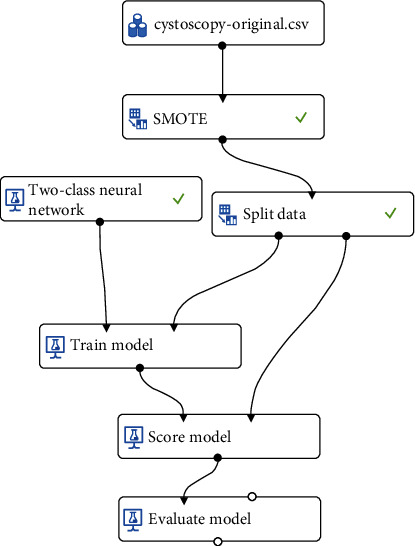
Neural network research flowchart.

An ANN on the Microsoft Azure Machine Learning Studio platform was used, and a two-class neural network was selected for the present research (Figure 1). Before normalized training data were input, the crucial parameters of the ANN (i.e., number of input neurons, number of hidden layers, number of neurons in the hidden layer, number of learning iterations, learning rate, and inertia factor) must first be determined because these parameters have crucial effects on the prediction error and convergence rate of the ANN. The parameters were tested multiple times to identify the optimal parameter combination with the fastest network convergence rate. The optimal parameter settings were as follows: 19 nodes in the neuron input layer, 2 fully connected hidden layers with 400 nodes in each layer, and a binary output layer (yes/no). In addition, the learning rate was set to 0.2; the learning iterations were set to 100, 200, 400, 800, and 1600; the initial weight was 0.2; and momentum was 0.5.

Variables were input to the binary logistic regression analysis in IBM SPSS to execute the training prediction model for establishing an optimized prediction model and determining the probability of the variables. The operation was repeated 20 times. Among the 19 variables, gender, antibiotic use within 1 month before the examination, and reasons for cystoscopy (i.e., difficulty urinating, urinary incontinence, bladder trabeculation, and bladder prolapse) were nonsignificant (*p* > 0.5). Therefore, these six nonsignificant variables were excluded. The remaining variables were subsequently reduced by eliminating variables for which *p* > 0.3, namely, hematuria, and tumor tracking. The predictive powers of the models including the 3 sets of variables (19, 13, and 11 variables) were compared.

## 3. Results

**Figure 2:**
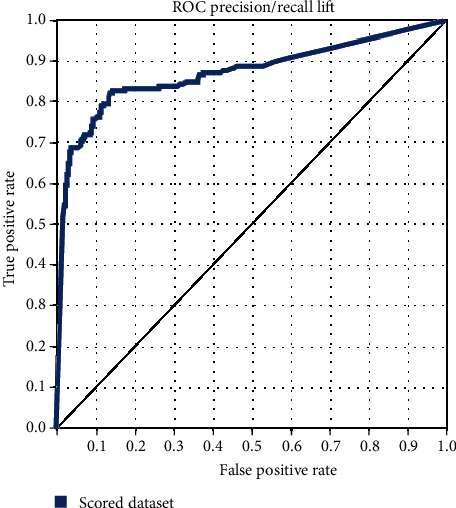
Two-Class Neural Network, ROC curve.

**Figure 3:**
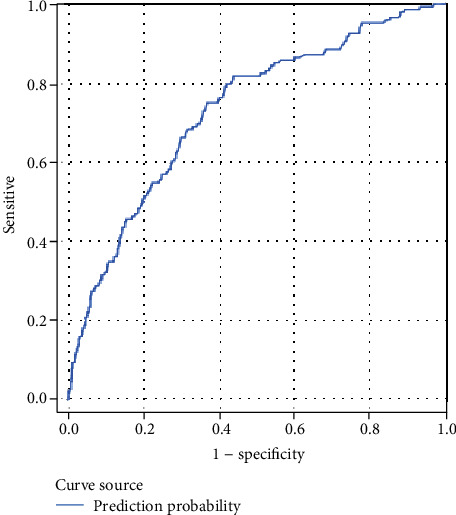
Logistic regression equation, ROC curve.

The ANN model had excellent predictive power and was effective in predicting UTI after cystoscopy in the diagnostic system. The optimal learning time for the ANN prediction model was 800 iterations. The accuracy, sensitivity, specificity, false positive rate (FPR), false negative rate (FNR), and area under the receiver operating characteristic (ROC) curve (AUC) were 85%, 80%, 88%, 11%, and 19%, and >0.8, respectively, indicating satisfactory predictive power (Figure 2). Moreover, 1600 iterations did not achieve the optimal prediction and instead reduced the accuracy and sensitivity (Table 3). In the logistic regression prediction model with all 19 variables (Table 4), the accuracy, sensitivity, specificity, FPR, FNR, and AUC were 91%, 2%, 99%, 0.26%, 97%, and 0.741, respectively (Figure 3). The logistic regression model predicted no infection in most patients, with a specificity of 99%. Nonetheless, the 2% sensitivity meant that the model could not predict infection in patients; therefore, it may have had a high misjudgment rate of actual infections. This phenomenon can cause an overfitting problem, which is undesirable in clinical medicine. Moreover, this phenomenon did not improve even after the number of variables was reduced to 13 and 11 (Table 5).

## 4. Discussion

Over the past decade, people have begun to use artificial intelligence in the medical field and gradually apply it to solve complex medical problems. The lack of comprehensive guidelines on antibiotic use and the low compliance rate of clinicians has led to antibiotic abuse. Therefore, this study explored whether the use of artificial intelligence can provide guidelines to reduce antibiotic abuse. In addition, a synthetic minority oversampling technique (SMOTE) was used to process unbalanced data by increasing them to 2392, with 894 positive data (37%) and 1498 negative data (63%). Furthermore, the splitting mode was applied to divide the dataset into training (80%) and test (20%) datasets. The ANN model predicted the probability of UTI caused by cystoscopy by using multiple learning iterations to evaluate the accuracy of the prediction results. When the number of learning iterations was 100, the accuracy, sensitivity, specificity, FPR, FNR, and AUC were 85%, 75%, 91%, 8%, 24%, and 0.875, respectively. At 200 iterations, they were 84%, 75%, 90%, 9%, 24%, and 0.875, respectively. At 400 iterations, they were 84%, 75%, 91%, 8%, 24%, and 0.867, respectively. At 800 iterations, they were 85%, 80%, 88%, 11%, 19%, and 0.868, respectively. Finally, at 1600 times, they were 84%, 79%, 88%, 11%, 20%, and 0.872, respectively (Table 3). An optimal parameter setting was obtained after multiple model tests, and the ROC curves for each set of learning iterations were similar, as shown in Figure 2.

According to the research analysis, 800 learning iterations in the ANN prediction model were optimal, demonstrating excellent predictive power. In addition, 1600 learning iterations did not achieve optimal prediction but reduced the accuracy and sensitivity. Ozkan et al. [7] compared four artificial intelligence methods often used in medical diagnostic systems, namely, decision tree, support vector machine, random forest, and ANN, for predictive analysis in the diagnosis of UTI. The respective accuracy, sensitivity, and specificity of the decision tree, support vector machine, random forest, and ANN models were 93.22%, 96.61%, 96.61%, and 98.30%; 95.55%, 97.77%, 95.55%, and 97.77%; and 85.71%, 92.85%, 100%, and 100% [7], which were similar to the results of the present study.

Regarding classification problems in research, class imbalance causes the classifier to generate biases during training, resulting in low prediction accuracy for minority class examples. For certain diseases that have an incidence of only 1%, machine learning methods might predict all cases to be normal, achieving an overall accuracy of 99%. However, the predictions were inaccurate for patients with the disease. This phenomenon is called overfitting.

To solve the aforementioned problems, Chawla et al. (2002) proposed SMOTE [8]. This method balances the class distribution by adding artificially synthesized minority samples to the data to reduce the possibility of overfitting and improve the generalization performance of the classifier on the test set. SMOTE has enabled the development of methods to resolve class imbalance and has made considerable contributions to new supervised learning paradigms, such as multilabel classification, incremental learning, semisupervised learning, and multi-instance learning [9]. SMOTE has become popular in the field of imbalance classification. In the present study, after 1647 medical records were input into the ANN model, SMOTE was used to process the imbalanced data, and then, the splitting mode was used to separate the data into training (80%) and test (20%) sets, as presented in Figure 1.

The 19 variables in the logistic regression prediction model were significantly different (Table 4). Considering the excessive number of variables that could affect the prediction model, those for which *p* > 0.5 (poor correlation) were eliminated, namely, age, antibiotic use within 1 month before the examination, difficulty urinating, urinary incontinence, bladder trabeculation, and bladder prolapse. By contrast, the variables were included in the ANN model because the model could manage multiple discrete variables.

The accuracy, sensitivity, specificity, FPR, FNR, and AUC of the 19-variable logistic regression model were 91%, 2%, 99%, 0.26%, 97%, and 0.741, respectively (Figure 3). Most patients in the logistic regression prediction model were predicted to not have an infection, achieving a specificity of 99%. However, the sensitivity of 2% indicates that the model cannot predict infection; in other words, the misjudgment rate of patients with infection patients was extremely high. This situation causes overfitting, which is undesirable in clinical medicine.

Yu, Ye, and Xiang (2016) compared an ANN with multivariate logistic regression to diagnose osteoporosis. The authors extracted characteristics from X-ray images and main clinical symptoms, and they evaluated characteristics related to osteoporosis according to quantitative standards. The sensitivity, specificity, and AUC were 94.5%, 96.9%, and 0.950 for the ANN model and 63.6%, 87.5%, and 0.870 for the logistic regression model, respectively. The ANN was effective in the diagnosis of osteoporosis [10]. In addition, Adavi (2016) compared the prediction accuracy of an ANN and multivariate logistic regression to simultaneously diagnose hypertension and diabetes (*n* = 12,000). Variables such as gender, cooking oil type, exercise habits, family history, age, smoking history, and obesity were input into the logistic regression and ANN models. The AUCs of the logistic regression and ANN models were 0.78 (*p* = 0.039) and 0.86 (*p* = 0.046), respectively. The findings indicated that the ANN model was the optimal predictive model for hypertension and diabetes because of its higher accuracy than that of the multivariate logistic regression model [11].

## 5. Conclusion

Clinicians generally determine whether patients require antibiotics on the basis of their experience or patient age. However, this method is prone to antibiotic abuse. The most common problem of artificial intelligence in medicine is unbalanced data because the majority of people are healthy but few have disease. This leads to overfitting problems in machine learning. Deviation may occur without proper data preprocessing, and the prediction model might predict the vast majority of patients to have no illness.

Numerous data preprocessing methods are available in machine learning, such as weight adjustment, multiple sampling, and data offset. These methods have been integrated into SMOTE in the Azure software, in which the learning model can be established through the drag-and-drop method. This method is easy for medical personnel to learn without a programming background.

Logistic regression analysis is the most common statistical tool because it has a high usage rate for discrete binary data. However, logistic regression models may have difficulty in prediction when encountering a large number of variables. On the other hand, the model may ignore potential factors if the variables are insufficient. In the present study, the logistic regression model had a large overfitting problem. By contrast, overfitting did not occur in the ANN model, which had higher sensitivity and specificity than did the logistic model.

Various studies have discussed the advantages and disadvantages of logistic regression and ANN models in medical applications. The present study revealed that the ANN model had superior results for the database used. Nevertheless, the analysis was limited to the database of a specific hospital. Therefore, future studies can include data from different medical institutions; in such studies, the logistic regression analysis may change.

## Figures and Tables

**Table 1:** Variables for collecting data.

Patient data	Cystoscopic indications	Cystoscopic findings
Gender	Voiding dysfunction	Cystitis
Age	Hematuria	BPH
Preexamination antibiotics in one month Preexamination UTI in one month	Stress urinary incontinence (SUI) Recurrent UTI Tumor survey	Diverticulum Trabeculation Blood clot Tumor
Residual urine > 100 ml		Stone Cystocele Urethral stricture

**Table 2:** Explanation of standardized coding of variable data.

Continuous variable	
Age (the age of the patient at the time of cystoscopy)	
*Category variable*	
Gender	1 is “male”, 0 is “female”
Preexamination antibiotics in one month	1 is “yes”, 0 is “no”
Preexamination UTI in one month	1 is “yes”, 0 is “no”
Residual urine > 100 ml	1 is “yes”, 0 is “no”
Voiding dysfunction	1 is “yes”, 0 is “no”
Hematuria	1 is “yes”, 0 is “no”
Stress urinary incontinence	1 is “yes”, 0 is “no”
Recurrent UTI	1 is “yes”, 0 is “no”
Tumor survey	1 is “yes”, 0 is “no”
Cystitis	1 is “yes”, 0 is “no”
BPH	1 is “yes”, 0 is “no”
Diverticulum	1 is “yes”, 0 is “no”
Trabeculation	1 is “yes”, 0 is “no”
Blood clot	1 is “yes”, 0 is “no”
Cystitis	1 is “yes”, 0 is “no”
Tumor	1 is “yes”, 0 is “no”
Cystocele	1 is “yes”, 0 is “no”
Urethral stricture	1 is “yes”, 0 is “no”

**Table 3:** Neural network model predictive analysis.

Number of learning iterations	100	200	400	800	1600
Sensitive (%)	75%	75%	75%	80%	79%
Specificity (%)	91%	90%	91%	88%	88%
FPR (%)	8%	9%	8%	11%	11%
FNR (%)	24%	24%	24%	19%	20%
Classification accuracy (%)	85.4%	84.5%	85.1%	85.1%	84.7%
AUC	0.875	0.875	0.867	0.868	0.872

**Table 4:** Variables in the logistic regression equation.

	*B*	S.E.	Wald	Significance	*Δ* odds (OR)	EXP(*B*) 95% confidence interval	
						Lower limit	Upper limit
Male	-0.012	0.191	0.004	0.949	0.988	0.680	1.435
Age	0.015	0.008	3.427	0.064	1.015	0.999	1.032
Preexamination UTI in one month	1.230	0.235	27.325	0.000	3.422	2.158	5.428
Preexamination antibiotics in one month	0.066	0.217	0.092	0.761	1.068	0.698	1.633
Residual urine > 100 ml	0.664	0.314	4.460	0.035	1.942	1.049	3.595
Voiding dysfunction	-0.118	0.296	0.158	0.691	0.889	0.497	1.589
SUI	0.013	0.547	0.001	0.982	1.013	0.347	2.959
Hematuria	-0.246	0.308	0.641	0.423	0.782	0.428	1.428
Recurrent UTI	0.372	0.402	0.853	0.356	1.450	0.659	3.191
Tumor survey	-0.311	0.334	0.870	0.351	0.732	0.381	1.409
Cystitis	0.267	0.258	1.075	0.300	1.306	0.788	2.164
BPH	0.503	0.209	5.809	0.016	1.654	1.099	2.491
Trabeculation	0.142	0.236	0.362	0.547	1.153	0.726	1.831
Diverticulum	0.416	0.332	1.578	0.209	1.517	0.792	2.905
Blood clot	1.155	0.354	10.647	0.001	3.173	1.586	6.348
Tumor	0.614	0.280	4.812	0.028	1.848	1.068	3.197
Stone	0.441	0.277	2.526	0.112	1.554	0.902	2.677
Urethral stricture	0.313	0.295	1.126	0.289	1.367	0.767	2.435
Cystocele	-18.630	8213.33	0.000	0.998	0.000	0.000	.
Constant	-4.058	0.701	33.547	0.000	0.017		

**Table 5:** Logistic regression model predictive analysis.

Number of variables	19	13	11
Sensitive (%)	2%	2%	2%
Specificity (%)	99%	99%	99%
FPR (%)	0.26%	0.20%	0.26%
FNR (%)	97%	97%	97%
Classification accuracy (%)	91%	91%	90.0%
AUC	0.741	0.731	0.706

## Data Availability

The data used to support the findings of this study are available from the corresponding author upon request.
